# Supporting People Data Linking Programme: Linked data research into the impact of housing-related support on use of healthcare services

**DOI:** 10.23889/ijpds.v8i2.2227

**Published:** 2023-09-14

**Authors:** Tony Whiffen, Kathryn Helliwell

**Affiliations:** 1 King's College London, London, United Kingdom

## Objectives

With an annual budget of over £120 million, the Welsh Government Supporting People Programme provided housing-related support to help vulnerable people to avoid homelessness and live as independently as possible. The SPDLP aims to determine the impact of the programme on other public services I.e. healthcare.

## Method

Establish data sharing agreements and acquire data for 21 out of 22 Local Authorities. Link the acquired Wales-wide sample of Supporting People data with other routine health and administrative datasets in the SAIL Databank. Examine the impact on subsequent interactions with other services (i.e. health, social care, housing options and pre-16 education). Create control groups and identify subgroups of clients to better quantify the effects of the Supporting People intervention. Compare service utilisation groups of clients and non-clients to inform future provision of public services.

## Results

Work to validate the SPDLP approach by applying the method from the earlier Supporting People Data Linking Feasibility Study (SPDLFS) to the most recent data has been completed. Results from this will be presented along with emerging findings related to repeat homelessness. The development of health economics approaches to evaluating the impact of the programme will also be presented.

## Conclusion

For most local authorities in Wales, we have developed a longitudinal linked dataset comprising details of people at risk of homelessness. This has successfully enabled statistical research and analysis to explore the wider impact of government programmes to address inequalities and answer research questions around homelessness.

